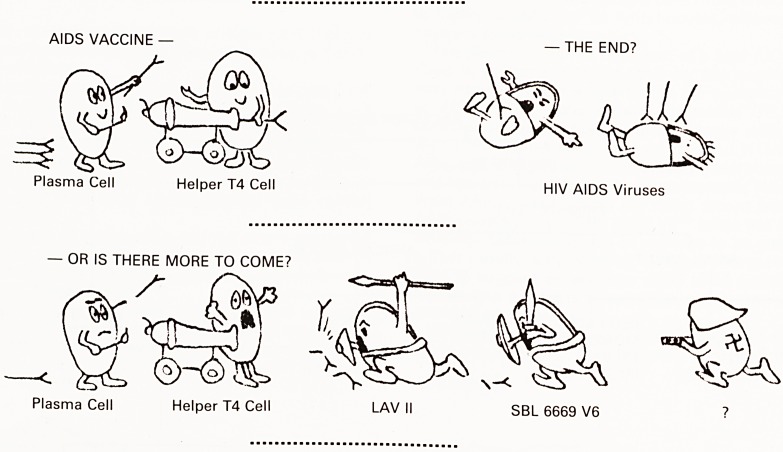# The Origin of AIDS

**Published:** 1987-08

**Authors:** J M A Smith

**Affiliations:** Medical Student, Bristol University


### The Origin of AIDS

**J M A Smith***
Medical Student, Bristol University

The Acquired Immune Deficiency Syndrome is a frightening new disease entity which has sprung, in five years, from obscurity to a major worldwide epidemic with a WHO estimate of 35000 cases worldwide, and extrapolations of antibody seropositivity rates suggest there to be between 1.5 and 2.5 million carriers in the USA and that 1% of the entire population of the African continent may be affected. The trend has been for an exponential increase in the number of individuals getting the disease (Fig. 1); a trend which, despite the various measures taken to counter the threat, seems unlikely to change in the near future. The disease has already overtaken homicide, suicide, accidents, and cancer as the most costly cause of loss of years of potential life, in Manhattan and San Francisco,[1] and it is a disease with one of the highest case fatality rates of any infectious disease of man.

Despite its recent arrival on the scene, the wheels of the scientific community are racing, with papers and letters published in almost every issue of the major scientific and medical journals, and the disease is the subject of daily press coverage and major government resource allocation. Yet there are some who would say that this is too little and too late, and it is certain that the significance of the disease was slow to be recognized, possible because of prejudicial attitudes to those populations first to be affected – homosexuals, intravenous drug abuses etc. It was only in the 1983 annual summary of the Morbidity and Mortality Weekly Report (MMWR), that it was transferred to the 'Notifiable Disease' category, though it is still not, in the strict sense of the word, notifiable, and to date there have been no reports on AIDS in the WHO Technical Report Series.

There are three main reporting mechanisms: (1) The Centres for Disease Control (CDC) in Atlanta, Georgia, which monitors the USA, and reports via the MMWR; (2) The Communicable Disease Surveillance Centre (CDSC) in London, which works with the Public Health Laboratory Service (PHLS), to coordinate reports from the UK; (3) The World Health Organisation (WHO) in Geneva, which is responsible for coordinating reports from all over the world, but which really only has accurate information for the USA, the UK and Europe.

In order for a new disease to be recognized it must have a strikingly unusual presentation, and a cluster of cases in a geographically well defined area, or in a specifically affected population group. AIDS had all these features. In June 1981 the CDC published a report on 5 cases of *Pneumocystis carinii pneumonia* (PCP) in three hospitals in Los Angeles; all were homosexuals, and all had signs of other opportunistic infections (OI).[2] This report was closely followed in the subsequent months by reports of Kaposi's sarcoma (KS) and PCP in homosexuals in California and New York dating retrospectively to 1979.[3,4]

It was detective work, such as that by Fannin *et al.*,[5] that was important in elucidating risk factors for the disease. In this case Fannin traced sexual contacts between 34 cases of KS and PCP in homosexuals in 9 different cities, so implicating rectal intercourse as an important mode of transmission of the disease.

Other at risk groups soon became apparent. In late 1981 intravenous drug abusers (IVDA) and Haitians were implicated and in early 1982 the first haemophiliac AIDS cases developed. The strongest predictive factor remained promiscuity, with a history of other sexually transmitted disease as a major prognostic factor. Whether this reflected lifestyle, or implicated cofactors as important in disease expression, or simply that the presence of genital lesions allowed easier inoculation of the causative organism, was not known.

A wide range of causative factors or cofactors were considered as to their aetiological importance, for instance cytomegalovirus, or inhalation of nitrite sphincter relaxants. Speculation abounded; indeed biological warfare experimentation was a suggestion that caused intense media interest, more than once.

In any disease, in order to obtain and usefully compare information, a surveillance definition is required. That for AIDS was a purely clinical one, since the cause was, at that time, unknown. Though a characteristic pattern of immunological profile was emerging with the observation of a decrease in T4 (helper) lymphocytes, often accompanied by a rise in T8 (suppressor) cells and other signs of immunodeficiency, tests were expensive and selective testing would have biased surveillance reports. The definition (which excluded the lymphadenopathy syndrome (LAS), AIDS related complex (ARC), prodromal or pre-AIDS, as it is variously called), demanded just two criteria – the presence of a reliably diagnosed disease at least moderately predictive of cellular immune deficiency, and the absence of any known underlying cause of that immune deficiency.

**Figure 1**

AIDS cases, by 6-month period of report to CDC – US, through November 22, 1986 (Data from: MMWR 34:775 1986; MMWR 35:17 1986; MMWR 35:424 1986; MMWR 35:735 1986)

In mid 1983, Montagnier's team at the Pasteur Institut, Paris, isolated a retrovirus from a patient with LAS, that they designated Lymphadenopathy Associated Virus (LAV).[6] The next year a similar virus, denoted HTLV III, was isolated from AIDS sufferers by an American team under Gallo.[7] LAV and HTLV III subsequently proved to be different isolates of the same species of virus, which is now called the Human Immunodeficiency Virus (HIV).

The isolation of the organism and the development of tests to detect antibodies in the serum of AIDS, pre-AIDS, and healthy seropositive subjects extends the range of surveillance for the disease by allowing the screening of at risk groups.

These serological tests have also allowed retrospective study of stored sera, which has shown the earliest occurrence of the virus in North America to have been around 1977–78,[8] and the earliest reference to AIDS in Europe was that of the retrospective diagnosis of the disease in a Cologne homosexual with KS and multiple OI in 1976.[9]

The majority of Europeans with the disease in these early years had connections with Africa – indeed of 23 Belgian patients with presentations consistent with AIDS, 22 had lived or worked in Zaire (the former Belgian Congo), and one had visited Burundi, in the previous 4 years.[10]

Similarly, many Haitian contacts of New York and Los Angeles homosexuals also are thought to have had connections with Zaire.

Retrospective reports of overt AIDS in Africa date back to 1976, to a Danish surgeon working in Zaire who developed multiple OI,[11] and to 1977, also in Zaire, where there was an outbreak of cryptococcal meningitis.[12] Serological studies using multiple tests indicated that the AIDS virus, or a related strain, was present in 1973 in Uganda,[13] and in at least one individual from central Africa in 1959![14]

These threads of information and the newly emerging picture of the sheer scale of AIDS in Africa, have lead to the hypothesis that the AIDS virus originated in, and spread from Africa (Fig 2).

HIV was originally labelled Human T cell Lymphotrophic Virus III, because it was the third retrovirus (RV) discovered to infect humans, and it was similar to the type C tumourigenic RV HTLVI (isolated in 1980 from patients with leukaemia) and to HTLVII (isolated from a patient with hairy cell leukaemia). It has also been shown to be related to the Lentivirinae – a group of animal viruses (Fig 3). It has been variously classed on morphological grounds both as a type C, and as a type D RV, but shows features more typical of the latter.

The recognition of an Acquired Immunodeficiency Disease in non human primates ten years ago, and more recently of outbreaks of such a disease in captive macaques in US primate research centres, lead to a search for a cause of this simian AIDS or SAIDS, and the discovery of a type C Simian T Lymphotropic Virus III (STLV III).[15]

Just as the discovery of the AIDS virus had been preceded by that of HTLV I, so the isolation of STLV III was preceded by that of a simian tumourigenic virus, STLV I, closely related to HTLV I. STLV III, which has also been isolated from healthy African Green Monkeys in the wild, infects a more limited range of species than STLV I and is presumed to be of more recent origin. It is similar to, but distinct from HIV (Fig 4).

Another RV, designated SAIDS-D RV, was implicated in the Washington primate research centre outbreaks of SAIDS, but was less selective in its tropisms for cells than HIV and STLV III in attacking B cells more effectively (HIV is highly specific for T4 lymphocytes, although it also appears to be neurotrophic).

The discovery earlier this year, of a new AIDS-causing virus in humans, called LAV II by Montagnier's team, may have provided a new link in the evolutionary chain of AIDS.[16] This virus closely resembles STLV III, and serologically cross reacts more strongly with this than with HIV. A third virus in man, labelled HTLV IV by its American discoverers, also closely resembles STLV III and has been isolated from asymptomatic prostitutes in West Africa,[17] but where the most recent isolate of an AIDS-causing virus, SBL 6669 V6[18] (after the State Bacteriology Laboratory), fits in remains to be seen.

This rapidly accumulating body of information would tend to suggest an ancestor AIDS virus endemic in primates, and suggests a transmission to man within the past few decades.

Research into the origin of the disease is not just an academic exercise. The discovery of SAIDS in non hu-

**Figure 2**

The Spread Of AIDS – A Hypothesis. (After Daniels: AIDS Questions and Answers, p.6 1986 and others)

(After White and Fenner: Medical Virology ch.8 1986)

Characteristics of the AIDS retrovirus (HIV)

Enveloped Type D virion 100–140 nm
Condensed cylindrical core
$Mg^{2+}$ dependant high MW reverse transcriptase
Single strand, diploid RNA genome 9500 bases
Heterogeneity of envelope gene (20–30%)
Budding from plasma membrane, long stalk
Cytocidal, not transforming for T4 lymphocytes
Viral DNA persists in integrated state
(White and Fenner: Medical Virology p. 587 1986)

**Figure 3.**

*The Retroviruses*

NON AIDS CAUSING
SAIDS CAUSING
AIDS CAUSING

(Modified after Watanebe *et al.* VIROLOGY 148:385 1986)

**Figure 4**

*The Evolution Of the AIDS Retrovirus*

man primates gives us a useful animal model for the study of AIDS and its pathogenesis. The similarity of behaviour, chemistry and morphology of the viruses together with the knowledge that HIV can be experimentally innoculated into primates, and conversely, monkey viruses can be transmitted to human cells in vitro, gives us a fair idea that we can be at least reasonably confident that the model is a good one.

This research is also a tool for the development of new antiviral drugs and of vaccines. The latter would be the ideal weapon in the fight against AIDS because of the fact that the virus incorporates its genome in the form of cDNA into the human T cells, and once infected, one may expect to be a persistent carrier, possibly for life. The development of vaccines would appear to be a viable proposition, since existing antibodies developed after primary infection with one strain of the AIDS virus, seem to prevent other common strains of HIV, which may differ by 20–30% of codons for the envelope gene, from subsequently infecting the same patient.

An American research team have gone further, in developing an inactivated viral vaccine which successfully prevented 6 macaques from developing SAIDS, where 5 of 6 controls were viraemic, and 4 developed SAIDS on subsequent innoculation with STLV III.[19]

Although HTLV IV so far has not been shown to cause disease (indeed we do not know how closely related to LAV II it is), it is unlikely that a live attenuated vaccine would be considered to be a safe, or ethical proposition, so a human vaccine would be aimed at the viral proteins of the envelope, perhaps at the receptor protein on the virus for T cells. However, due to the variability of the codons, the vaccine would have to be directed at relatively conserved regions of the proteins. Work is going on in the genetic engineering of the Vaccinia virus, so that this could express HIV envelope proteins on its surface, in a highly immunogenic way.

Assuming that an effective vaccine could be developed, would this be the end of the story? The recent discoveries of the new disease causing organisms LAV II and SBL 6669 V6 are important in that these viruses could escape detection by the existing serological screening tests, and that a vaccine against one virus might not protect against another.

I would like to thank Dr Sandy Macara for his encouragement and support.

### REFERENCES

1. CURRAN, J. W. *et al.* (1985) The epidemiology of AIDS – current status and future prospects. *Science.* **299**, 1352.
2. CDC. (1981) *Pneumocystis* pneumonia – Los Angeles. *MMWR.* **30**, 250.
3. CDC. (1981) KS and PCP among homosexual men – New York City and California. *MMWR.* **30**, 305.
4. CDC. (1981) Follow up on KS and PCP. *MMWR.* **30**, 409.
5. FANNIN, S. L. *et al.* (1982) A cluster of KS and PCP among homosexual residents of LA and Orange county. *MMWR.* **31**, 305.
6. BARRE-SINOUS, F. *et al.* (1983) Isolation of a T lymphotrophic retrovirus from a patient at risk from AIDS. *Science.* **220**, 868.
7. GALLO, R. C. *et al.* (1984) Frequent detection and isolation of HTLV III from patients with AIDS. *Science.* **224**, 500.
8. MADHOK, R. *et al.* (1985) HTLV III antibody in sequential plasma samples from haemophiliacs (1974–1984). *Lancet.* **1**, 524.
9. STERRY, W. *et al.* (1983) KS, pancytopaenia and multiple infections in a homosexual (Cologne 1976). *Lancet.* **1**, 924.
10. CLUMECK, N. *et al.* (1983) AIDS in African patients. *N. Eng. J. Med.* **310**, 492.
11. BYCBERG, I. C. *et al.* (1983) AIDS in a Danish surgeon (Zaire 1976). *Lancet.* **1**, 925.
12. VANDEPITTE, J. *et al.* (1983) AIDS and Cryptococcus (Zaire 1977). *Lancet.* **1**, 925.
13. SAXINGER, W. C. (1985) Evidence for HTLV III exposure in Uganda before 1973. *Science.* **227**, 1036.
14. NAHMIAS, A. J. *et al.* (1986) Evidence for human infection with an HTLV III/LAV like virus in central Africa 1959. *Lancet.* **1**, 1279.
15. DANIEL, M. D. *et al.* (1985) Isolation of a T cell lymphotrophic HTLV III like a retrovirus from macaques. *Science.* **228**, 1201.
16. CLAVEL, F. *et al.* (1986) Isolation of a new human retrovirus from African patients with AIDS. *Science.* **233**, 343.
17. KANKI, P. J. *et al.* (1986) New human T lymphotrophic retrovirus related to STLV III AGM. *Science.* **232**, 238.
18. THOMAS, C. Researchers find a third virus. *The Times*, 22.11.86, p1.
19. GROOPMAN, J. E. (1986) Retroviral vaccines: How close is C to D? *Nature.* **323**, 489.

## Figures and Tables

**Figure 1 f1:**
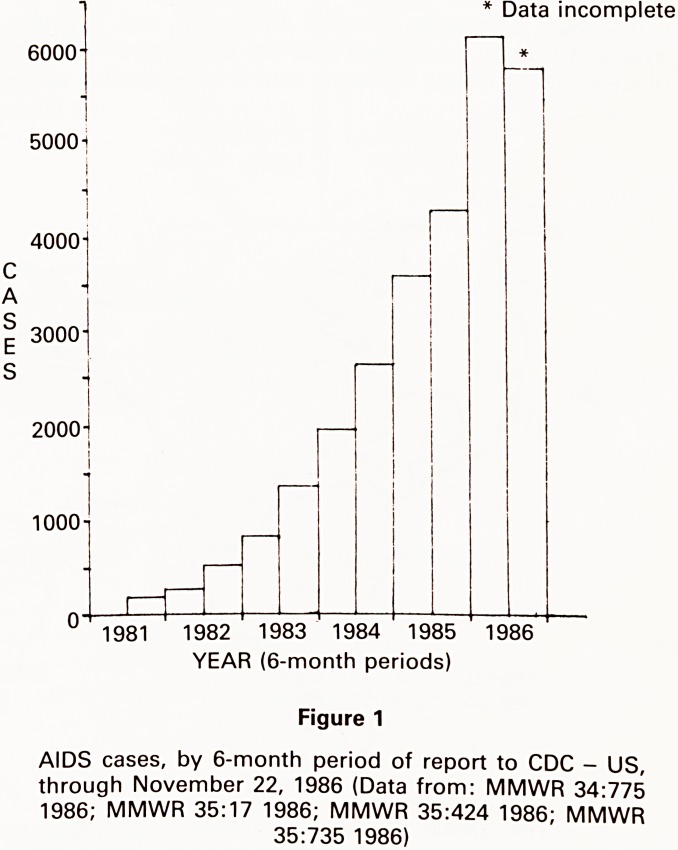


**Figure 2 f2:**
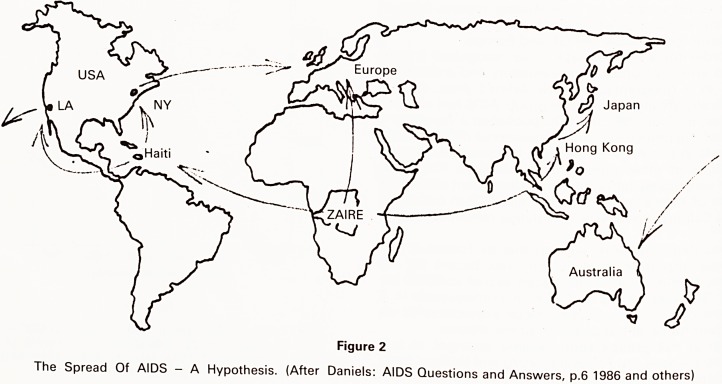


**Figure 3. f3:**
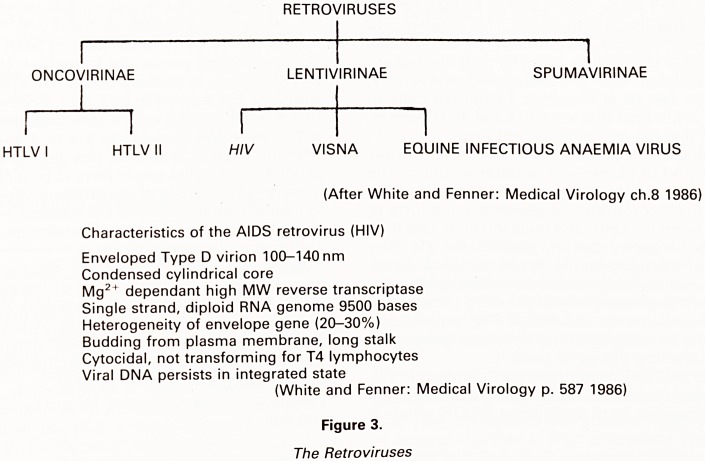


**Figure 4 f4:**
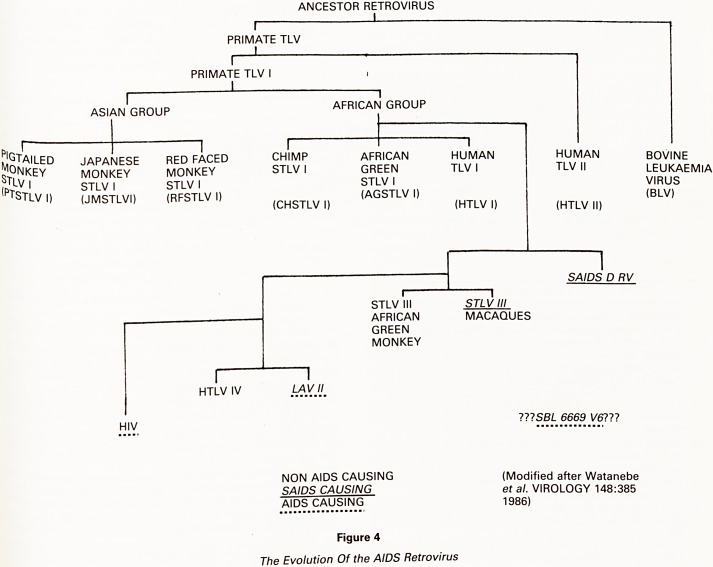


**Figure f5:**